# Supplementary material for: COZOID: contact zone identifier for visual analysis of protein-protein interactions
Source: BMC Bioinformatics. 2018 Apr 6;19:125. doi: 10.1186/s12859-018-2113-6 (PMC5889581; doi:10.1186/s12859-018-2113-6)
Supplement: Supplementary file 2 — Software build. Executable binary file of the software tool. (ZIP 118,784 kb) [file 12859_2018_2113_MOESM2_ESM.zip › cozoid/java/docs/beansbinding-1.2.1-doc.zip!/org/jdesktop/beansbinding/ext/BeanAdapterFactory.html]

BeanAdapterFactory (Beans Binding)


---


|  |  |  |
| --- | --- | --- |
| **Overview** \| **Package** \| **Class** \| **Use** \| **Tree** \| **Deprecated** \| **Index** \| **Help** | |  |
| PREV CLASS   **NEXT CLASS** | **FRAMES**    **NO FRAMES**     **All Classes** |  |
| SUMMARY: NESTED \| FIELD \| CONSTR \| METHOD | DETAIL: FIELD \| CONSTR \| METHOD |  |


---


## org.jdesktop.beansbinding.ext Class BeanAdapterFactory

```
java.lang.Object
  org.jdesktop.beansbinding.ext.BeanAdapterFactory
```

---

``` public final class BeanAdapterFactory extends java.lang.Object ```

---

**Constructor Summary**

`BeanAdapterFactory()`


| **Method Summary** | |
| --- | --- |
| `static java.lang.Object` | `getAdapter(java.lang.Object source, java.lang.String property)` |
| `java.lang.Object` | `getAdapter0(java.lang.Object source, java.lang.String property)` |
| `static java.util.List<java.beans.PropertyDescriptor>` | `getAdapterPropertyDescriptors(java.lang.Class<?> type)` |

**Methods inherited from class java.lang.Object**

`clone, equals, finalize, getClass, hashCode, notify, notifyAll, toString, wait, wait, wait`

**Constructor Detail**

### BeanAdapterFactory

```
public BeanAdapterFactory()
```


**Method Detail**

### getAdapter

```
public static java.lang.Object getAdapter(java.lang.Object source,
                                          java.lang.String property)
```

---


### getAdapterPropertyDescriptors

```
public static java.util.List<java.beans.PropertyDescriptor> getAdapterPropertyDescriptors(java.lang.Class<?> type)
```

---


### getAdapter0

```
public java.lang.Object getAdapter0(java.lang.Object source,
                                    java.lang.String property)
```


---


|  |  |  |
| --- | --- | --- |
| **Overview** \| **Package** \| **Class** \| **Use** \| **Tree** \| **Deprecated** \| **Index** \| **Help** | |  |
| PREV CLASS   **NEXT CLASS** | **FRAMES**    **NO FRAMES**     **All Classes** |  |
| SUMMARY: NESTED \| FIELD \| CONSTR \| METHOD | DETAIL: FIELD \| CONSTR \| METHOD |  |


---
